# 4,6-Dibromo-2-[(*E*)-(4-{[(*E*)-3,5-dibromo-2-hy­droxy­benzyl­idene]amino}­butyl)­imino­meth­yl]phenol

**DOI:** 10.1107/S1600536812028863

**Published:** 2012-06-30

**Authors:** Hadi Kargar, Reza Kia, Amir Adabi Ardakani, Muhammad Nawaz Tahir

**Affiliations:** a Department of Chemistry, Payame Noor University, PO Box 19395-3697 Tehran, I. R. of IRAN; b Department of Chemistry, Science and Research Branch, Islamic Azad University, Tehran, Iran; c Department of Physics, University of Sargodha, Punjab, Pakistan

## Abstract

The asymmetric unit of the title compound, C_18_H_16_Br_4_N_2_O_2_, comprises half the molecule, which is located adjacent to an inversion centre at the mid-point of the central C—C bond of the butane-1,4-diamine segment. There are two intra­molecular O—H⋯N hydrogen bonds making *S*(6) ring motifs. In the crystal, mol­ecules are linked by pairs of weak C—H⋯Br inter­actions into chains along [101], which include *R*_2_^2^(8) ring motifs. These chains are further linked by C—H⋯O hydrogen bonds, forming a three-dimensional network.

## Related literature

For standard bond lengths, see: Allen *et al.*, (1987 ▶). For hydrogen-bond motifs, see: Bernstein *et al.* (1995 ▶). For related Schiff base ligands, see: Kargar *et al.* (2011 ▶); Kia *et al.* (2010 ▶).
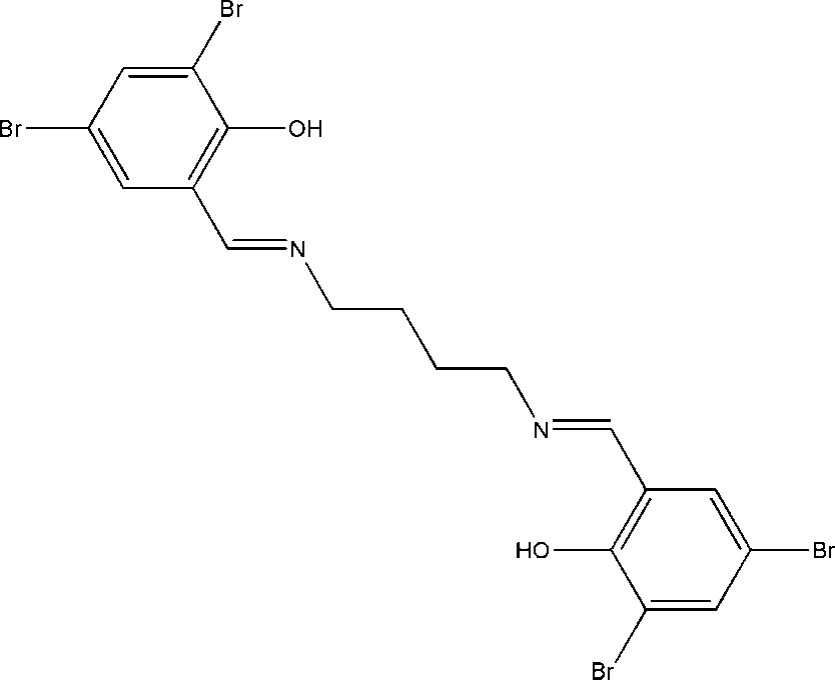


## Experimental

### 

#### Crystal data

C_18_H_16_Br_4_N_2_O_2_
*M* *_r_* = 611.97
Orthorhombic, 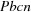

*a* = 15.9537 (12) Å
*b* = 12.8784 (10) Å
*c* = 9.5566 (6) Å
*V* = 1963.5 (2) Å^3^
*Z* = 4
Mo *K*α radiation
μ = 8.21 mm^−1^
*T* = 291 K
0.35 × 0.14 × 0.12 mm

#### Data collection

Bruker SMART APEXII CCD area-detector diffractometer
Absorption correction: multi-scan (*SADABS*; Bruker, 2005 ▶) *T*_min_ = 0.161, *T*_max_ = 0.439
15023 measured reflections
2164 independent reflections
1381 reflections with *I* > 2σ(*I*)
*R*_int_ = 0.069

#### Refinement

*R*[*F* ^2^ > 2σ(*F* ^2^)] = 0.035
*wR*(*F* ^2^) = 0.074
*S* = 0.99
2164 reflections
118 parameters
H-atom parameters constrained
Δρ_max_ = 0.74 e Å^−3^
Δρ_min_ = −0.57 e Å^−3^

### 

Data collection: *APEX2* (Bruker, 2005 ▶); cell refinement: *SAINT* (Bruker, 2005 ▶)’; data reduction: *SAINT*; program(s) used to solve structure: *SHELXS97* (Sheldrick, 2008 ▶); program(s) used to refine structure: *SHELXL97* (Sheldrick, 2008 ▶); molecular graphics: *SHELXTL* (Sheldrick, 2008 ▶); software used to prepare material for publication: *SHELXTL* and *PLATON* (Spek, 2009 ▶).

## Supplementary Material

Crystal structure: contains datablock(s) global, I. DOI: 10.1107/S1600536812028863/su2461sup1.cif

Structure factors: contains datablock(s) I. DOI: 10.1107/S1600536812028863/su2461Isup2.hkl

Supplementary material file. DOI: 10.1107/S1600536812028863/su2461Isup3.cml

Additional supplementary materials:  crystallographic information; 3D view; checkCIF report

## Figures and Tables

**Table 1:** Hydrogen-bond geometry (Å, °)

*D*—H⋯*A*	*D*—H	H⋯*A*	*D*⋯*A*	*D*—H⋯*A*
O1—H1⋯N1	0.72	1.92	2.574 (4)	151
C6—H6⋯O1^i^	0.93	2.56	3.469 (5)	165
C4—H3⋯Br2^ii^	0.93	2.96	3.849 (4)	161

Symmetry codes: (i) 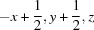; (ii) 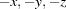.

**Fig. 1.**
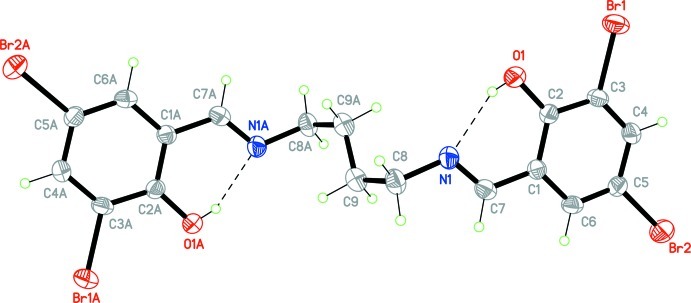
The molecular structure of the title molecule, showing 40% probability displacement ellipsoids and the atomic numbering [symmetry code for suffix A = -x, -y, -z].

**Fig. 2.**
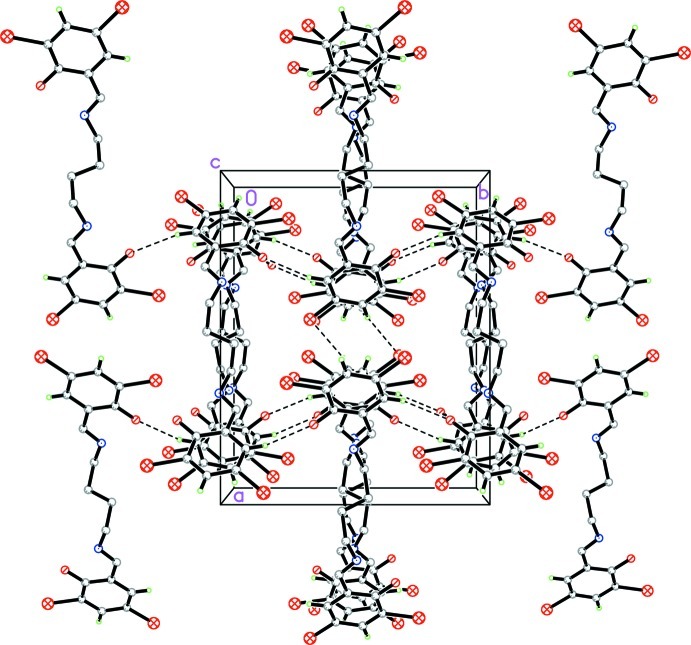
The crystal packing of the title compound viewed along the c axis, showing linking of molecules through C—H···O and weak C—H···Br interactions (dashed lines; see Table 1 for details). Only the H atoms involved in hydrogen bonding are shown.

## supplementary crystallographic information

### Comment

In continuation of our work on the crystal structures of Schiff base ligands (Kargar *et al.*, 2011; Kia *et al.*, 2010), we synthesized and analysed the crystal structure of the title compound.

The asymmetric unit of the title compound, Fig. 1, comprises half of a potential tetradentate Schiff base ligand. The molecule is located about an inversion centre which is situated at the centre of the central C9-C9A bond of the 1,4-butane-diamine segment. The bond lengths (Allen *et al.*, 1987) and angles are within the normal ranges. The intramolecular O—H···N hydrogen bonds make *S(6)* ring motifs (Table 1 and Fig. 1; Bernstein *et al.*, 1995).

In the crystal, molecules are linked by pairs of weak C—H···Br interactions to form chains along direction [101] which include *R^2^_2_(8)* ring motifs (Table 1 and Fig. 2). These chains are further linked by C—H···O interactions (Table 1) to form a three-dimensional network.

### Experimental

The title compound was synthesized by adding 3,5-dibromosalicylaldehyde (2 mmol) to a solution of butylenediamine (1 mmol) in ethanol (30 ml). The mixture was refluxed with stirring for 30 min. The resultant solution was filtered. Yellow needle-like crystals of the title compound, suitable for *X*-ray structure determination, were obtained by recrystallization from ethanol on slow evaporation of the solvents at room temperature over several days.

### Refinement

The OH H atom was located in a difference Fourier map and constrained to ride on the parent O atom with U_iso_(H) = 1.5U_eq_(O). The C-bound H-atoms were included in calculated positions and treated as riding atoms: C—H = 0.93 and 0.97 Å for CH and CH_2_ H-atoms, respectively, with U_iso_(H) = 1.2U_eq_(C).

### Crystal data

**Table:**

C_18_H_16_Br_4_N_2_O_2_	*F*(000) = 1176
*M**_r_* = 611.97	*D*_x_ = 2.070 Mg m^−^^3^
Orthorhombic, *P**b**c**n*	Mo *K*α radiation, λ = 0.71073 Å
Hall symbol: -P 2n 2ab	Cell parameters from 2045 reflections
*a* = 15.9537 (12) Å	θ = 3.3–27.5°
*b* = 12.8784 (10) Å	µ = 8.21 mm^−^^1^
*c* = 9.5566 (6) Å	*T* = 291 K
*V* = 1963.5 (2) Å^3^	Needle, yellow
*Z* = 4	0.35 × 0.14 × 0.12 mm

### Data collection

**Table:**

Bruker SMART APEXII CCD area-detector diffractometer	2164 independent reflections
Radiation source: fine-focus sealed tube	1381 reflections with *I* > 2σ(*I*)
Graphite monochromator	*R*_int_ = 0.069
φ and ω scans	θ_max_ = 27.2°, θ_min_ = 2.0°
Absorption correction: multi-scan (*SADABS*; Bruker, 2005)	*h* = −20→20
*T*_min_ = 0.161, *T*_max_ = 0.439	*k* = −16→16
15023 measured reflections	*l* = −11→11

### Refinement

**Table:**

Refinement on *F*^2^	Primary atom site location: structure-invariant direct methods
Least-squares matrix: full	Secondary atom site location: difference Fourier map
*R*[*F*^2^ > 2σ(*F*^2^)] = 0.035	Hydrogen site location: inferred from neighbouring sites
*wR*(*F*^2^) = 0.074	H-atom parameters constrained
*S* = 0.99	*w* = 1/[σ^2^(*F*_o_^2^) + (0.0269*P*)^2^ + 1.1226*P*] where *P* = (*F*_o_^2^ + 2*F*_c_^2^)/3
2164 reflections	(Δ/σ)_max_ < 0.001
118 parameters	Δρ_max_ = 0.74 e Å^−^^3^
0 restraints	Δρ_min_ = −0.57 e Å^−^^3^

### Special details

**Table:**

Geometry. Bond distances, angles etc. have been calculated using the rounded fractional coordinates. All su's are estimated from the variances of the (full) variance-covariance matrix. The cell esds are taken into account in the estimation of distances, angles and torsion angles
Refinement. Refinement of F^2^ against ALL reflections. The weighted R-factor wR and goodness of fit S are based on F^2^, conventional R-factors R are based on F, with F set to zero for negative F^2^. The threshold expression of F^2^ > 2sigma(F^2^) is used only for calculating R-factors(gt) etc. and is not relevant to the choice of reflections for refinement. R-factors based on F^2^ are statistically about twice as large as those based on F, and R- factors based on ALL data will be even larger.

### Fractional atomic coordinates and isotropic or equivalent isotropic displacement parameters (Å^2^)

**Table:**

	*x*	*y*	*z*	*U*_iso_*/*U*_eq_	
Br1	0.13066 (3)	−0.24496 (3)	0.17601 (5)	0.0577 (2)	
Br2	0.05752 (3)	0.17551 (3)	0.07666 (5)	0.0549 (2)	
O1	0.25622 (17)	−0.15166 (19)	0.3764 (3)	0.0456 (9)	
N1	0.3337 (2)	−0.0051 (2)	0.5060 (3)	0.0374 (11)	
C1	0.2230 (2)	0.0284 (3)	0.3459 (4)	0.0320 (11)	
C2	0.2109 (2)	−0.0776 (3)	0.3154 (4)	0.0324 (11)	
C3	0.1493 (2)	−0.1035 (3)	0.2181 (4)	0.0359 (11)	
C4	0.1036 (2)	−0.0293 (3)	0.1493 (4)	0.0373 (12)	
C5	0.1186 (2)	0.0745 (3)	0.1788 (4)	0.0373 (12)	
C6	0.1761 (2)	0.1035 (3)	0.2756 (4)	0.0349 (11)	
C7	0.2860 (2)	0.0599 (3)	0.4468 (4)	0.0360 (12)	
C8	0.3979 (2)	0.0338 (3)	0.6028 (4)	0.0433 (14)	
C9	0.4818 (3)	0.0488 (3)	0.5281 (5)	0.0537 (17)	
H1	0.28180	−0.12680	0.42830	0.0680*	
H3	0.06320	−0.04820	0.08400	0.0450*	
H6	0.18440	0.17350	0.29520	0.0420*	
H7	0.29150	0.13000	0.46860	0.0430*	
H8A	0.40490	−0.01510	0.67920	0.0520*	
H8B	0.37970	0.09950	0.64210	0.0520*	
H9A	0.47400	0.09730	0.45150	0.0650*	
H9B	0.52140	0.07960	0.59310	0.0650*	

### Atomic displacement parameters (Å^2^)

**Table:**

	*U*^11^	*U*^22^	*U*^33^	*U*^12^	*U*^13^	*U*^23^
Br1	0.0699 (3)	0.0317 (2)	0.0716 (3)	−0.0021 (2)	−0.0215 (3)	−0.0097 (2)
Br2	0.0590 (3)	0.0417 (2)	0.0641 (3)	0.0064 (2)	−0.0152 (2)	0.0100 (2)
O1	0.0460 (17)	0.0348 (14)	0.0561 (18)	0.0052 (13)	−0.0166 (14)	−0.0036 (13)
N1	0.0298 (17)	0.0403 (18)	0.042 (2)	−0.0008 (16)	−0.0012 (16)	−0.0022 (15)
C1	0.027 (2)	0.0309 (19)	0.038 (2)	−0.0025 (17)	0.0029 (19)	0.0002 (17)
C2	0.029 (2)	0.0313 (19)	0.037 (2)	0.0010 (17)	0.0004 (18)	0.0008 (17)
C3	0.037 (2)	0.0267 (18)	0.044 (2)	−0.0035 (18)	0.0021 (19)	−0.0057 (17)
C4	0.035 (2)	0.038 (2)	0.039 (2)	−0.0024 (19)	−0.0080 (19)	−0.0016 (18)
C5	0.033 (2)	0.034 (2)	0.045 (2)	0.0023 (18)	−0.0035 (19)	0.0043 (18)
C6	0.038 (2)	0.0258 (18)	0.041 (2)	−0.0018 (17)	0.005 (2)	−0.0003 (17)
C7	0.037 (2)	0.034 (2)	0.037 (2)	−0.0042 (18)	0.003 (2)	−0.0058 (18)
C8	0.038 (2)	0.053 (3)	0.039 (2)	−0.003 (2)	−0.006 (2)	−0.006 (2)
C9	0.043 (3)	0.049 (3)	0.069 (3)	−0.003 (2)	−0.013 (3)	−0.011 (2)

### Geometric parameters (Å, º)

**Table:**

Br1—C3	1.889 (4)	C4—C5	1.387 (5)
Br2—C5	1.896 (4)	C5—C6	1.355 (5)
O1—C2	1.331 (5)	C8—C9	1.529 (6)
O1—H1	0.7200	C9—C9^i^	1.485 (6)
N1—C7	1.265 (5)	C4—H3	0.9300
N1—C8	1.468 (5)	C6—H6	0.9300
C1—C2	1.409 (5)	C7—H7	0.9300
C1—C6	1.395 (5)	C8—H8A	0.9700
C1—C7	1.451 (5)	C8—H8B	0.9700
C2—C3	1.393 (5)	C9—H9A	0.9700
C3—C4	1.370 (5)	C9—H9B	0.9700
			
C2—O1—H1	107.00	C8—C9—C9^i^	113.8 (3)
C7—N1—C8	118.4 (3)	C3—C4—H3	121.00
C2—C1—C6	119.9 (3)	C5—C4—H3	121.00
C2—C1—C7	120.2 (3)	C1—C6—H6	120.00
C6—C1—C7	119.9 (3)	C5—C6—H6	120.00
O1—C2—C3	120.3 (3)	N1—C7—H7	119.00
C1—C2—C3	117.8 (3)	C1—C7—H7	119.00
O1—C2—C1	121.9 (3)	N1—C8—H8A	109.00
Br1—C3—C2	118.9 (3)	N1—C8—H8B	109.00
C2—C3—C4	121.9 (4)	C9—C8—H8A	109.00
Br1—C3—C4	119.1 (3)	C9—C8—H8B	109.00
C3—C4—C5	118.9 (3)	H8A—C8—H8B	108.00
Br2—C5—C6	120.7 (3)	C8—C9—H9A	109.00
C4—C5—C6	121.4 (3)	C8—C9—H9B	109.00
Br2—C5—C4	117.9 (3)	H9A—C9—H9B	108.00
C1—C6—C5	120.0 (4)	C9^i^—C9—H9A	109.00
N1—C7—C1	121.9 (3)	C9^i^—C9—H9B	109.00
N1—C8—C9	111.1 (3)		
			
C8—N1—C7—C1	177.7 (3)	O1—C2—C3—C4	−177.5 (3)
C7—N1—C8—C9	−94.4 (4)	C1—C2—C3—Br1	−179.4 (3)
C6—C1—C2—O1	177.7 (3)	C1—C2—C3—C4	2.5 (5)
C6—C1—C2—C3	−2.3 (5)	Br1—C3—C4—C5	−178.9 (3)
C7—C1—C2—O1	−0.3 (5)	C2—C3—C4—C5	−0.8 (5)
C7—C1—C2—C3	179.7 (3)	C3—C4—C5—Br2	177.6 (3)
C2—C1—C6—C5	0.4 (5)	C3—C4—C5—C6	−1.2 (5)
C7—C1—C6—C5	178.4 (3)	Br2—C5—C6—C1	−177.4 (3)
C2—C1—C7—N1	2.7 (5)	C4—C5—C6—C1	1.4 (5)
C6—C1—C7—N1	−175.2 (3)	N1—C8—C9—C9^i^	−62.9 (5)
O1—C2—C3—Br1	0.6 (5)	C8—C9—C9^i^—C8^i^	−180.0 (3)

Symmetry code: (i) −*x*+1, −*y*, −*z*+1.

### Hydrogen-bond geometry (Å, º)

**Table:**

*D*—H···*A*	*D*—H	H···*A*	*D*···*A*	*D*—H···*A*
O1—H1···N1	0.72	1.92	2.574 (4)	151
C6—H6···O1^ii^	0.93	2.56	3.469 (5)	165
C4—H3···Br2^iii^	0.93	2.96	3.849 (4)	161

Symmetry codes: (ii) −*x*+1/2, *y*+1/2, *z*; (iii) −*x*, −*y*, −*z*.
